# Polypoidal Choroidal Vasculopathy Diagnosis and Neovascular Activity Evaluation Using Optical Coherence Tomography Angiography

**DOI:** 10.1155/2021/1637377

**Published:** 2021-11-16

**Authors:** Georges Azar, Vivien Vasseur, Corinne Lahoud, Catherine Favard, Flore De Bats, Isabelle Cochereau, Amélie Yachvitz, Martine Mauget-Faÿsse

**Affiliations:** ^1^Rothschild Ophthalmological Foundation, Anterior Segment Department, 25 Rue Manin, 75940 Paris Cedex 19, France; ^2^Rothschild Ophthalmological Foundation, Center of Clinical Investigation, 25 Rue Manin, 75940 Paris Cedex 19, France; ^3^Holy Spirit University of Kaslik (USEK), Faculty of Medicine, Kaslik, Lebanon; ^4^Pole Vision Center, Val-d'Ouest Clinics, 39, Chemin de la Vernique, 69130 Écully, France

## Abstract

**Purpose:**

To examine choroidal neovascularization (CNV) characteristics in patients with exudative age-related macular degeneration (AMD) and polypoidal choroidal vasculopathy (PCV), using swept-source optical coherence tomography angiography (SS-OCTA), and investigate agreement with OCT B-scan, fundus fluorescein angiography (FFA), and indocyanine green angiography (ICGA) by two different examiners.

**Methods:**

This is a retrospective multicentric study that involved patients with a history of AMD and PCV. *Examiner A*, who had access to OCTA, B-scan OCT, FFA, and ICGA imaging, had to differentiate between AMD and PCV, study the activity of AMD using Coscas' criteria (active vs. quiescent), and categorize PCV subtypes, while *examiner B* had only access to OCTA. Then, the diagnostic concordance was assessed between both examiners.

**Results:**

A total of 27 patients (11 females (40.7%) and 16 males (59.3%), *P* = 0.231) were included in the analysis. Among those, 13 patients presented with neovascular AMD and 14 patients with PCV. There were 92.3% of correct answers regarding appropriate diagnosis and lesion characterization among AMD patients, against 61.5% of correct answers among PCV patients. The overall interrater reliability agreement between examiners, using Cohen's kappa coefficient (*κ*) was 0.70 (0.5082-0.8916). Disagreement was found with *one active AMD* misdiagnosed as inactive AMD, *three inactive PCV* misdiagnosed as inactive AMD, and *one inactive PCV* misdiagnosed as active AMD.

**Conclusion:**

SS-OCTA alone remains limited in some specific phenotypes of PCV, which suggests the ongoing role of B-scan OCT associated with FFA and ICGA in the diagnosis of these conditions.

## 1. Introduction

Polypoidal choroidal vasculopathy (PCV) is a clinical entity, with its own characteristics, distinctive from neovascular age-related macular degeneration (AMD). It is a vascular disease of the choroid characterized by an abnormal branching vascular network (BVN) and aneurysmal dilations called polyps. These subretinal polypoidal vascular lesions may be associated with serous and hemorrhagic pigment epithelial detachments (PED) [1–4] PCV is the most impactful subtype of AMD. In fact, it provides a marker of anti-VEGF resistance and may affect therapeutic planning for treatment-naïve eyes [5].

PCV can be classified clinically into 2 groups: *quiescent* and *active*. *Quiescent PCV* is characterized by the presence of one or several polyp(s) in the absence of any intraretinal or subretinal fluid or hemorrhage. It is asymptomatic and treatment is usually not needed. On the other hand, *exudative or active PCV* manifests with exudation due to leakage from active polyps and/or BVN. This latter group may be further classified into exudative, hemorrhagic, or mixed. Clinically, the exudation comes in the form of either serous PED, subretinal fluid, serous macular detachment, subretinal lipid exudation, or intraretinal fluid, whereas hemorrhagic PCV presents with subretinal or subretinal pigment epithelium (RPE) hemorrhage [6].

Indocyanine green angiography (ICGA) is the gold standard for the diagnosis of CNV and PCV. It provides the clinician with a powerful adjunctive tool in establishing differential diagnosis between PCV and atypical AMD. However, this modality may not be routinely used and requires intravenous dye injection, which can arise allergic or renal complications in some cases [3].

Among recent retinal imaging technologies, optical coherence tomography angiography (OCTA) allows noninvasive visualization of retinal and choroidal structure without requiring dye injection. It creates a motion contrast by differentiating between moving cells in the vasculature and static surrounding tissue. This contrast is created by calculating the decorrelation of signal amplitude from repeated B-scans at the same cross section, which enables a 3-D visualization of retinal and choroidal vasculature. OCTA has the ability to track changes in the choroidal blood vessel flow and can highlight the presence of CNV in multiple pathologies, particularly in AMD [4, 5].

Nevertheless, studies on the efficacy of OCTA in diagnosing PCV remain sparse: in one study, Fujita et al. concluded that this new modality is an ideal and safe option for the diagnosis of PCV and could replace ICGA in the near future [7].

On the other hand, Inoue et al. suggested that OCTA could help in the management of polypoidal diseases but has limitations that preclude it from replacing ICGA [8]. Moreover, Huang et al. concluded that OCTA may be useful for the detection and classification of PCV and that ICGA should be reserved for cases in which OCTA is negative but PCV is highly suspected [9]. Overall, OCTA greatly contributes to the detection and the evaluation of AMD but seems less reliable in PCV detection, where two factors must be considered separately: the polyps and the BVN [8, 9].

The purpose of this study is to determine the agreement between SS-OCTA and multimodal imaging with fluorescein fundus angiography (FFA), ICGA, and OCT-B-scan in the diagnosis of PCV and evaluation of neovascular activity. It is also aimed at assessing the ability of SS-OCTA to differentiate alone between AMD and PCV and at studying PCV's neovascular activity.

Finally, we also aim to establish the presence of biomarkers of PCV on OCTA and to determine whether Coscas' criteria for the evaluation of AMD on OCTA are applicable to PCV/BVN activity.

## 2. Methods

### 2.1. Study Design

This is a retrospective observational study that was conducted at the Rothschild Ophthalmological Foundation and at the Odeon Ophthalmological Center, Paris, France. A series of 14 consecutive patients presenting with PCV and 13 other patients with neovascular AMD, admitted between January 2018 and January 2019, were analyzed. Approval was obtained from the Research and Development Department and the Institutional Ethics Committee as a retrospective study does not require informed consent. The study adhered to the tenets of the Declaration of Helsinki.

### 2.2. Data Collection

The medical charts of consecutive patients presenting with AMD and PCV were reviewed. Data concerning demographic information, the presence of systemic comorbidities, and clinical examinations were collected. This included measurement of best-corrected visual acuity (BCVA) assessed with the Early Treatment Diabetic Retinopathy Study- (ETDRS-) like charts at an initial testing distance of 4 m, slit-lamp examination, direct and indirect ophthalmoscopy, and Goldmann applanation tonometry. Moreover, analyses from color fundus photograph images, FFA, ICGA, B-scan OCT, and morphologic analysis using OCTA were also obtained.

### 2.3. Morphologic Correlation Study and Diagnosis Criteria

AMD neovascular activity, PCV subtype analysis, and BVN diagnosis criteria were all assessed from SS-OCTA obtained using a deep ranging imaging (DRI) OCT Triton. Three fifty-micron slabs were shifted and analyzed around the appropriate layer over and under the RPE. OCTA software within a commercially available device that operates at 70.000 A-scans per second to acquire OCTA volumes was used for all patients, allowing visualization of both retinal and choroidal flow and structure, based on split-spectrum amplitude decorrelation angiography. At first, the initial diagnosis made at clinic and the neovascular activity of AMD and PCV were assessed using multimodal imaging with FFA, ICGA, and SS-OCTA (*examiner A*). Then, SS-OCTA images were analyzed by a second independent expert operator (*examiner B*), blinded to all other multimodality images, which determined whether patients had AMD or PCV and assessed neovascular activity. Both examiners were retina specialists.

#### 2.3.1. CNV Activity Criteria in AMD

**Figure 1:**
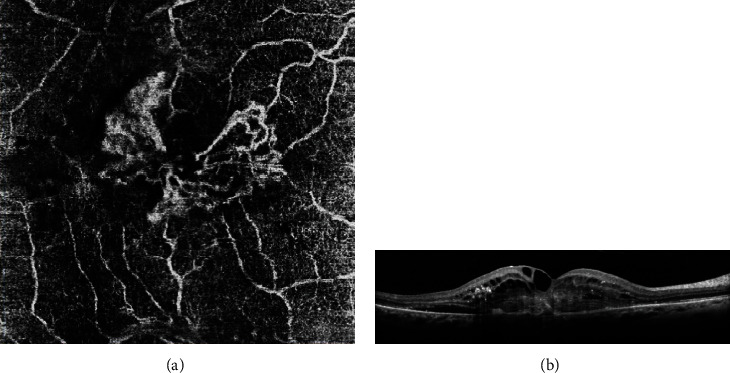
Active choroidal new vessel on OCT-A (a) according to Coscas' criteria: wheel shape, dense branching pattern, anastomosis and loops, peripheral arcade, sprouting capillaries, and black peripheral halo. B-scan OCT showing activity of retrofoveal CNV (b).

**Figure 2:**
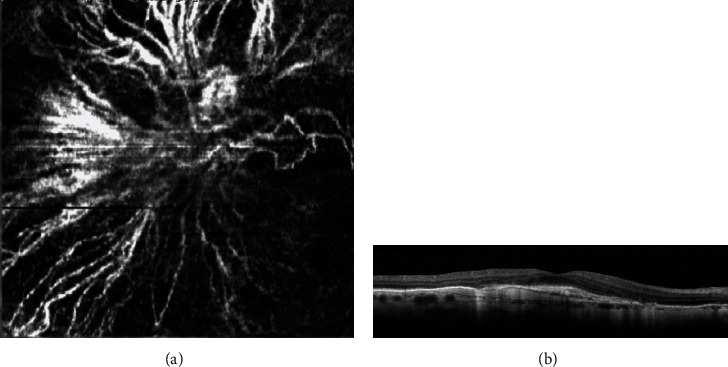
Quiescent (long-lasting) choroidal new vessel on OCT-A (a) according to Coscas' criteria: long and filamentous shape, loose radial branching pattern, dead-tree aspect, and lack of loops. B-scan OCT showing fibrotic CNV scar (b).

CNV activity in AMD patients was assessed using Coscas' criteria in exudative AMD using OCTA. According to his classification, *active lesions* present with at least one of the following criteria (Figures 1(a) and 1(b)): a wheel shape, a dense branching pattern, anastomosis and loops, a peripheral arcade, sprouting capillaries, and/or peripheral halo [10, 11]. On the other hand, *quiescent lesions* (Figures 2(a) and 2(b)) have a long and filamentous shape, loose branching patterns, a dead-tree aspect, and lack of loops, peripheral arcade, and halo.

#### 2.3.2. PCV Subtypes and BVN Analysis

PCV diagnosis was based on Tan et al.'s classification, which differentiates PCV into 3 subtypes by performing a combination of FFA and ICGA: *type A* is defined by the presence of a polyp with interconnecting channels on ICGA, *type B* is a polyp with BVN without leakage on FFA, and *type C* is a polyp with BVN that presents with late leakage on FFA [12]. Criteria for the establishment of BVN diagnosis included shape of the lesion, branching pattern, the presence or absence of anastomoses and loops, morphology of vessel termini, and the presence or absence of a perilesional hypointense halo [10]. Moreover, neovascular activity criteria for polyps included the presence of black holes, hypo/hypersignals, loops, and halos. Finally, BVN was described according to OCTA characteristics as dense, very dense (bush-shape), loose (dead-tree shape), pseudopod-like, and anastomoses.

### 2.4. Statistical Analysis

Statistical analysis was performed using commercially available software (SPSS version 20.0, Inc., Chicago, Illinois). Interrater reliability for categorical items between examiners was assessed using Cohen's kappa coefficient (*κ*). This test is a more robust measure than simple percent agreement calculation, as *κ* takes into account the possibility of the agreement occurring by chance. The statistical significance was set at *P* < 0.05.

## 3. Results

### 3.1. Study Sample

A total of 27 patients (11 females (40.7%) and 16 males (59.3%), *P* = 0.231) were included in the analysis. Among those, 13 patients (6 females and 7 males) presented with neovascular AMD and 14 patients (5 females and 9 males) with PCV. The mean patient age was 81 ± 4.9 years (range, 73-86 years) for the AMD group and 72 ± 6.1 years (range, 66-83 years) for the PCV group, which included 13 Caucasians and 1 Asian. In this same latter group, 10 patients presented with active lesions and 4 had inactive lesions. Diagnosis evaluation by examiner B using only OCTA imaging revealed positive AMD diagnosis in 13 out of 13 patients (100%) and positive PCV diagnosis in 10 out of 14 patients (71.4%).

### 3.2. PCV Morphological Analysis with OCTA

All polyps were located above Bruch's membrane. Two polyps presented with a halo, whereas 3 out of 14 polyps presented with a hyperreflective round structure surrounded with a hypointense halo. The mean PCV choroidal thickness was 232.6 ± 122.1 *μ*m.

### 3.3. BVN Morphological Analysis with OCTA

**Figure 3:**
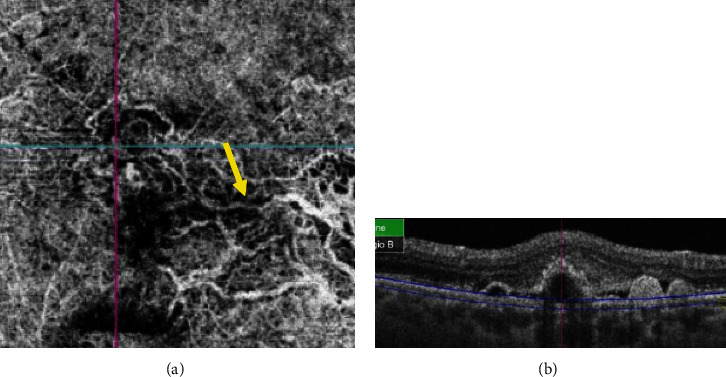
OCTA image (a) showing a branching vascular network (yellow arrow) located between RPE and Bruch's membrane as shown in B-scan OCT (b).

**Figure 4:**
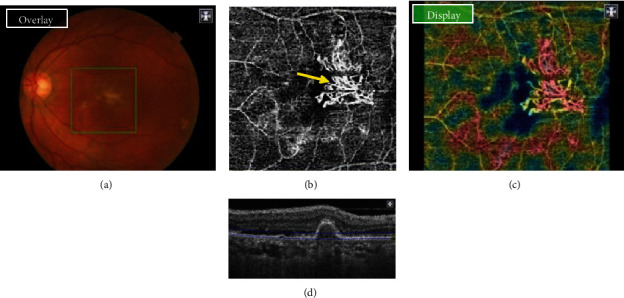
Color picture (Topcon, TRC) (a) showing an active yellowish lesion located in the foveal region. OCTA (b) showing a type C PCV (yellow arrow) with a branching vascular network of a “bush-shape” aspect. Multiple high-density cross-sectional swept-source “en face” OCT (c), showing a choroidal vascularity map and highlighting the “bush-shape” aspect of BVN. B-scan OCT (d) showing an active PCV lesion adjacent to serous retinal detachment.

In all cases (100%), the BVN was located between the RPE and Bruch's membrane (Figure 3). This BVN was *loose* in 7 out of 14 patients (50%) and *dense* in the others (50%). In the former (*loose BVN*) group, 6 patients presented with active PCV (85.8%) and 1 patient with inactive PCV (14.2%) whereas in the latter (*dense BVN*) group, 4 patients presented with active PCV (57.1%), among which 2 lesions had a bush-shape aspect (Figure 4) and 3 patients had inactive PCV (42.9%).

### 3.4. Interrater Reliability Analysis of Disease Activity

**Figure 5:**
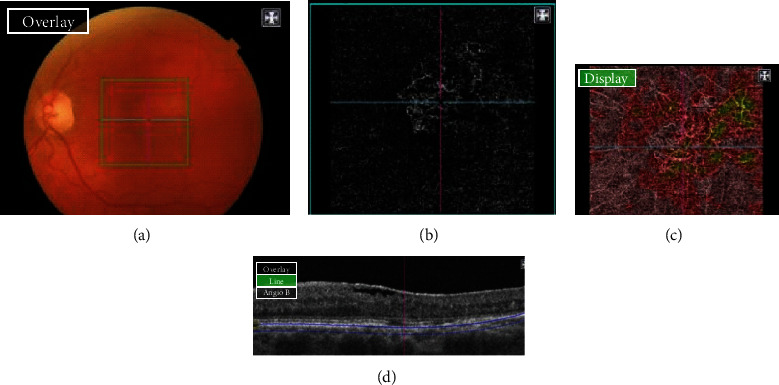
Inactive PCV misdiagnosed with active AMD by examiner B. Color picture (Topcon, TRC) (a) showing the absence of CNV activity while OCTA (b) showing a dense branching pattern with anastomosis and loops, recalling the presence of activity according to Coscas' criteria, diagnosed as active AMD. “En face” OCT (c), recalling the presence of a “tangled filamentous” neovessel branching vascular network. B-scan OCT (d) showing no sign of CNV activity.

When trying to determine an agreement in the diagnosis and neovascular activity of PCV and AMD between *examiner A* (who had access to OCTA and all other multimodal imaging) and *examiner B* (with only OCTA), there were 92.3% of correct answers regarding appropriate diagnosis and lesion characterization among AMD patients, against only 61.5% of correct answers among PCV patients. Finally, the overall interrater reliability agreement between both examiners, using Cohen's kappa coefficient (*κ*), was 0.70 (0.5082-0.8916). Disagreement was found with *one active AMD* misdiagnosed as inactive AMD, *three inactive PCV* misdiagnosed as inactive AMD, and *one inactive PCV* (Figure 5) misdiagnosed as active AMD.

## 4. Discussion

PCV is a disease characterized by polypoidal-like dilated choroidal vessels linked to interconnecting channels or a BVN [12]. Although ICGA remains the gold standard in the diagnosis by highlighting the polyp lesions, this technique requires a professional operator, is considered to be invasive, and is not commonly used in many parts of the world. Because of those limitations, other imaging investigations are currently a popular research area for the diagnosis of PCV. Recently, some studies have reported the detection rate of polyps and BVN lesions by OCTA, a noninvasive technique that studies the microvasculature activity of the retina and the choroid, and studied the sensitivity and specificity of the diagnosis of PCV from wet-AMD patients. However, the results among the majority of those studies remain inconsistent. Therefore, the main purpose of this study was to determine the agreement between (SS)-OCTA and multimodal imaging with FFA, ICGA, and B-scan OCT evaluation in the diagnosis of PCV and in the evaluation of neovascular activity and to highlight if SS-OCTA is able to determine alone those entities.

First, our results show that all polyps were located above Bruch's membrane, which is comparable with the results of a study done by Chi et al. on 47 patients with PCV [13]. Two polyps presented with halo, which is similar to a study by Srour et al. where 3 out of 12 patients with PCV presented with a round hyperreflective structure surrounded with a hypointense halo [14]. On the other hand, BVN was located between the RPE and Bruch's membrane in all cases, which is concordant with a study done by Tomiyasu et al. [15]. Interestingly, the presence of hyposignal halo around the polyp was accompanied by a late leakage as shown with ICGA, which suggests a certain correlation between hyposignal halo and neovascular activity. Whether the presence of this hyposignal halo shown on SS-OCTA may be a biomarker of polyp activity requires further investigation on larger series to fully prove it.

A meta-analysis done recently by Wang et al. revealed a high diagnostic detection rate of polyps and BVN by OCTA in PCV, and the detection rate of BVN was higher than that of polyps [16]. However, although OCTA has greatly facilitated the detection of BVN by providing a thorough description of their structure [17], polypoidal lesions were poorly visualized on en face SS-OCTA and some reports suggest that they may be missed with this technique because of undetectable blood flow [18, 19]. However, observation from other studies that use SS-OCTA has suggested a higher sensitivity in the detection of polypoidal lesions [20]. In fact, SS-OCTA uses a laser light source with a longer wavelength than that of SD-OCTA (1060 mm vs. 840 mm). Consequently, because of less sensitivity roll-off happening under the RPE, structural and angiographic images appear superior to those of SD-OCT [21–23]. When trying to determine an agreement between SS-OCTA and multimodal imaging, our findings show that there were 92.3% of correct answers regarding appropriate diagnosis and lesion characterization among AMD patients, against 61.5% of correct answers among PCV patients. Although this technique requires a learning curve to fully assimilate and validate all the structural findings of the retinal and choroidal microvasculature, it provides very high-quality images in the detection of PCV.

OCTA has greatly facilitated the detection of BVNs and has provided detailed descriptions of their structure [18, 19]. Representations like “coral bush shape,” “dead-tree shape,” “pseudopod-like,” and “anastomoses” have been widely described. Besides, various manifestations of polyp lesions in PCV were also reported in studies in which terms like “black holes,” “cluster-like structures,” “round structures with increased or decreased flow characteristics,” and “halos” have been used [21–23]. Nevertheless, none of those terms regarding the shape of BVN and PCV was correlated with the disease activity or response to treatment, especially when categorizing PCV into its type A, B, or C. In our study, assessment of disease activity using only ICGA and OCTA was not constantly congruent. In fact, while OCTA suggested an absence of neovascular activity, for instance, in PCV with “loose” BVN, FA and B-scan OCT did show in some cases late leakage and exudative signs, which suggests that this type of BVN is not necessarily a sign of inactivity. On the other hand, our observations show that the presence on OCTA of a “coral bush-shape” BVN is a positive sign of active type C PCV.

Finally, when assessing OCTA images in patients treated with anti-Vascular Endothelial Growth Factor (VEGF), we found that BVN presenting in the form of “pseudopod-like extrusions” tended to have more neovascular activity during follow-up, suggesting that this specific type of BVN may represent a higher risk of recurrences, which might influence the therapeutic approach during treatment decisions. Our findings suggest that Coscas' CNV activity criterion grid on OCTA can only be applicable on some types of PCV, especially the active type C PCV.

This study has limitations. First, it includes a relatively small sample of patients, which is due to the low incidence of PCV in the population. Besides, in order to be able to achieve the main objective of the study, which was to determine the agreement between SS-OCTA and multimodal imaging with FFA, ICGA, and OCT-B-scan in the diagnosis of PCV and evaluation of neovascular activity, we only included patients with PCV and exudative AMD that have undergone all the above exams. This could have a certain selection bias. In addition, this is a purely retrospective observational study with no prospective comparison between two cohorts, which also could make the results relatively biased. On the other hand, OCTA is a relatively new ophthalmology technology that is continuously being promoted in clinical settings, which makes its capacity to detect PCV heterogeneous in the literature.

In conclusion, although SS-OCTA seems to be superior in detection of PCV activity when compared with SD-OCTA, it remains limited in some specific phenotypes, which suggests the ongoing role of ICGA in the diagnosis of this pathology. Categorizing PCV into types A, B, and C and specifying the description of BVN seem to advocate in some cases neovascular activity and risk of recurrences. However, more research is still needed to explore the clinical value of SS-OCTA in the diagnosis, natural history, and prognosis of PCV. Larger studies remain essential to confirm our findings and to determine whether each phenotype described of BVN and PCV correlates with its neovascular activity and response to treatment.

## Data Availability

The authors declare to have full access to the data analysis and take responsibility for the integrity and accuracy of the presented results.
